# Avian Antimicrobial Host Defense Peptides: From Biology to Therapeutic Applications

**DOI:** 10.3390/ph7030220

**Published:** 2014-02-27

**Authors:** Guolong Zhang, Lakshmi T. Sunkara

**Affiliations:** 1 Department of Animal Science, Oklahoma State University, Stillwater, OK 74078, USA; 2 Department of Biochemistry and Molecular Biology, Oklahoma State University, Stillwater, OK 74078, USA; 3 Department of Physiological Sciences, Oklahoma State University, Stillwater, OK 74078, USA

**Keywords:** host defense peptides, antimicrobial resistance, antibiotic alternatives, chicken

## Abstract

Host defense peptides (HDPs) are an important first line of defense with antimicrobial and immunomoduatory properties. Because they act on the microbial membranes or host immune cells, HDPs pose a low risk of triggering microbial resistance and therefore, are being actively investigated as a novel class of antimicrobials and vaccine adjuvants. Cathelicidins and β-defensins are two major families of HDPs in avian species. More than a dozen HDPs exist in birds, with the genes in each HDP family clustered in a single chromosomal segment, apparently as a result of gene duplication and diversification. In contrast to their mammalian counterparts that adopt various spatial conformations, mature avian cathelicidins are mostly α-helical. Avian β-defensins, on the other hand, adopt triple-stranded β-sheet structures similar to their mammalian relatives. Besides classical β-defensins, a group of avian-specific β-defensin-related peptides, namely ovodefensins, exist with a different six-cysteine motif. Like their mammalian counterparts, avian cathelicidins and defensins are derived from either myeloid or epithelial origin expressed in a majority of tissues with broad-spectrum antibacterial and immune regulatory activities. Structure-function relationship studies with several avian HDPs have led to identification of the peptide analogs with potential for use as antimicrobials and vaccine adjuvants. Dietary modulation of endogenous HDP synthesis has also emerged as a promising alternative approach to disease control and prevention in chickens.

## 1. Introduction

Host defense peptides (HDPs), also known as antimicrobial peptides, constitute a large group of small peptides that have been discovered in virtually all forms of life [1,2,3]. Natural HDPs are generally positively charged and comprised of less than 100 amino acid residues with amphipathic properties. HDPs represent an important first-line of defense particularly in those species whose adaptive immune system is lacking or primitive. Most HDPs are encoded by distinct genes, and a large number of structurally different HDPs normally exist in a single species. A majority of HDPs are strategically synthesized in the host phagocytic and mucosal epithelial cells that regularly encounter the microorganisms from the environment. Synthesized initially as precursors, HDPs are generally processed by host proteases to release mature peptides upon infection and inflammation [1,2].

Mature HDPs are broadly active against Gram-negative and Gram-positive bacteria, mycobacteria, fungi, viruses, and even cancerous cells [1,2]. Relying primarily on the physical membrane-lytic mechanisms, HDPs kill bacteria with a low risk of triggering resistance [4]. Additionally, HDPs were recently found to interact specifically with several membrane-bound or intracellular receptors with a profound ability to modulate the host response to inflammation and infection [5,6,7]. Because of antimicrobial and immunomodulatory activities, HDPs are being actively explored for antimicrobial therapy particularly against drug-resistant microbes [8]. A number of HDPs have been found with preferential expression in the male reproductive tract and several are linked to sperm maturation and might have potential for infertility treatment [9,10].

## 2. Classification of HDPs

HDPs are structurally diversified. Based on the secondary structure, HDPs are classified into α-helical, β-sheet, αβ, and non-αβ families [1,2,3,11]. In general, α-helical peptides are amphipathic, often with a bend around the central region disrupting an otherwise fairly perfect cylindrical structure. The β-sheet HDPs are usually formed by the presence of disulfide bonds with amphipathic patches scattered on the surface. The αβ-peptides consist of both α-helical and β-sheet structures, whereas non-αβ HDPs are free of α-helical and β-sheet structures, often unstructured, and rich in proline, arginine or histidine residues.

### 2.1. Cathelicidins

**Figure 1:**
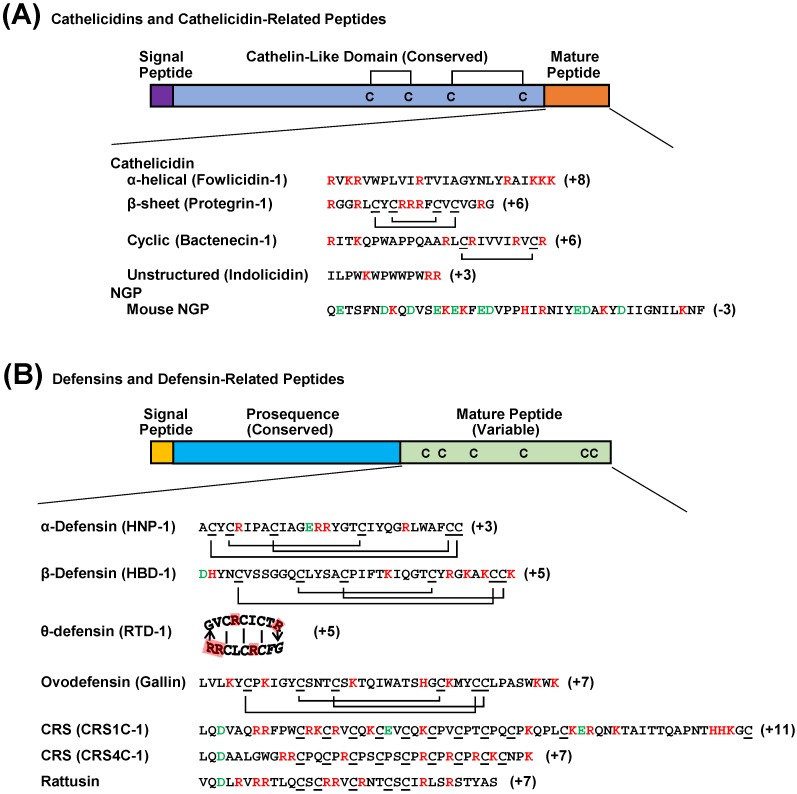
Schematic drawing of vertebrate cathelicidin and defensin precursor peptides. (**A**) Cathelicidins and cathelicidin-related peptides known as NGPs are highly conserved in the cathelin-like domain that contains two disulfide bridges. Unlike cathelicidins whose C-terminal segments are highly variable across species and proteolytically cleaved from the cathelicidin-like domain to become biologically active, NGPs are conserved throughout the entire sequence and functionally active without being processed. (**B**) The defensin family includes classical α-, β-, and θ-defensins with indicated disulfide bonds as well as four subfamilies of defensin-related peptides with unknown disulfide bonding patterns. Avian-specific ovodefensins contain six cysteines but with a different spacing pattern from that of classical defensins. Rodent-specific CRS1C, CRS4C, and rattusin also exist with 11, 9, and 5 cysteine residues, respectively, that presumably form intermolecular disulfide bonds. Positively and negatively charged amino acids are indicated in red and green, respectively.

Among an ever increasing number of HDPs that have been reported, two major families, namely cathelicidins and defensins, exist in vertebrate animals [12,13,14] (Figure 1). Cathelicidins have been reported in a variety of vertebrate species including fish, amphibians, reptiles, birds, and mammals [12,15]. Multiple cathelicidins normally exist in each species, except for euarchontogliers (e.g., primates, rabbits, and rodents) and carnivorans (e.g., cats and dogs). Cathelicidins are named for the presence of a cathelin-like domain in the N-terminal region of the peptide precursor (Figure 1). Although the signal peptide and cathelin-like domain of cathelicidins are extremely conserved across species, mature peptide sequences at the C-terminal region are highly diversified even within a species. Neutrophilic granule proteins (NGPs) are a group of cathelicidin-related HDPs that have been reported in rabbits, rodents, and many other mammalian species [16,17,18]. Although they apparently have evolved from cathelicidins, NGPs are conserved throughout the entire sequence, including the C-terminal region. Given rabbit NGP, also known as p15, is biologically active without proteolytic cleavage [19], it is possible that other NGPs may not be processed to become biologically active.

### 2.2. Defensins

Vertebrate defensins are further classified into three subfamilies including α-, β-, and θ-defensins that are characterized by the presence of six cysteines with different spacing and bonding patterns (Figure 1) [9,13,14]. The α-defensins are mammalian-specific with a C1-C6, C2-C4, and C3-C5 cysteine-bridging pattern, whereas β-defensins are universal in vertebrates with a C1-C5, C2-C4, and C3-C6 bridging pattern. The θ-defensins, on the other hand, have only been discovered in primates, with a pseudogene present in the human genome [20]. The six cysteines of θ-defensins form a cyclic structure by a head-to-tail ligation of two truncated α-defensins [20]. Three additional subfamilies of α-defensin-related peptides have also been found in rodents [21,22]. Two groups of cryptdin-related sequence (CRS) peptides, namely CRS1C and CRS4C, appear to exist only in mice with 11 and nine cysteines, respectively [21]. A unique rat-specific rattusin was recently reported to consist of five cysteines [22]. Although rodent defensin-related sequences are located within the α-defensin gene cluster and highly similar to α-defensins in the signal and pro-sequences, the disulfide bridging patterns of the C-terminal mature peptides are totally different among them (Figure 1). Albeit with no reported tertiary structures, these defensin-related peptides are likely to form homo- or hetero-dimers or oligomers because of the presence of an odd number of cysteines. Another group of β-defensin-related molecules, recently classified as ovodefensins, appear to be specific in birds [23]. Albeit with six cysteines in the C-terminal mature region forming a disulfide bonding pattern identical to β-defensins [24], avian ovodefensins consist of a different cysteine spacing pattern (Figure 1).

## 3. Discovery of Avian HDPs

Both the cathelicidin and defensin families of HDPs exist in birds [25,26]. However, NGPs appear to be specific to mammals and no NGP-like cathelicidins have been reported in any avian species. Only β-defensins have been discovered in birds, and no α- or θ-defensins exist. Rodent-specific rattusin or CRS peptides are also absent in birds. However, ovodefensins are uniquely present in several avian species [23]. Excellent reviews are available on the general knowledge of avian HDPs [27,28], and this review will focus on their biology and therapeutic applications, with more emphasis on the similarities and differences between avian and mammalian HDPs.

### 3.1. Avian Cathelicidins

Four distinct cathelicidin genes have been reported in birds. The first two avian cathelicidins (CATH1 and CATH2) were reported in chickens in 2004 and 2005, respectively [29,30]. The same two peptides, together with a third chicken cathelicidin (CATH3), were also independently reported as fowlicidin 1–3 [18]. The fourth chicken cathelicidin, known as CATH-B1, was discovered to be preferentially expressed in the bursa of Fabricius [31], a specialized organ for hematopoiesis and B cell development in birds. All four chicken cathelicidins were found to cluster densely together within a 7.5-kb distance toward one end of chromosome 2 [18,31]. All four chicken cathelicidin genes adopt a 4-exon, 3-intron structure, typical for a mammalian cathelicidin. The first three exons encode the 5′-untranslated region, signal peptide, and a majority of the cathelin-like domain, while the last exon encodes primarily the mature peptides, in addition to the 3′-untranslated region [18,31]. Chicken CATH1–3 share similar exon sizes and a typical cathelin-like domain with mammalian cathelicidins, whereas CATH-B1 consists of an alternatively spliced and unusually large first exon and an uncharacteristic cathelin-like domain [31].

**Figure 2:**
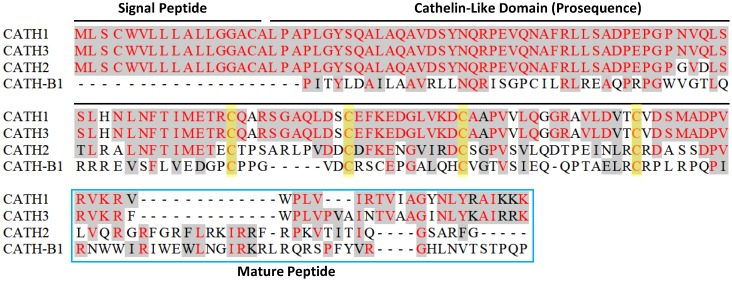
Amino acid sequence alignment of four chicken cathelicidins. Conserved sequences are shaded and identical residues are in red. Dashes are created to maximize the alignment. Each cathelicidin precursor consists of a conserved signal peptide sequence, a cathelin-like domain, and a variable C-terminal mature peptide sequence. Four cysteines in the cathelin-like domain are highlighted in yellow. Note that an N-terminal, 117-amino acid segment of CATH-B1 was omitted for clarity.

Alignment of four chicken cathelicidin peptide sequences revealed that they are similar to each other and also homologous to mammalian cathelicidins in the signal peptide and cathelin-like domain (Figure 2). Among them, CATH1 and CATH3 are most closely related with >90% identity throughout the entire sequence, while CATH-B1 is a distant member, sharing only 40% with CATH1 (Figure 2). The orthologs of chicken CATH1–3 were also recently reported in several other avian species such as the common quail and common pheasant [32,33]. With recent completion of genome sequencing for the turkey, mallard duck, rock dove (*Columba livia*), ground tit (*Pseudopodoces humilis*), saker falcon (*Falco cherrug*), Peregrine Falcon (*Falco peregrinus*), and budgerigar (*Melopsittacus undulatus*), a number of avian cathelicidin sequences have been predicted and become available in GenBank.

### 3.2. Avian β-Defensins

More than a dozen unique β-defensin genes are present, and no α- or θ-defensins exist in birds. The first two avian β-defensins, known as gallinacins 1–2, were isolated in 1994 from chicken heterophils [34], an equivalent of mammalian neutrophils. The turkey orthologs of gallinacins 1–2 were also independently purified from heterophil granules later in the same year [34]. With the completion of the chicken genome sequencing, a large number of additional chicken β-defensin genes were independently reported by Lynn, *et al.* [29] and Xiao, *et al.* [35], respectively. Because of different numbering systems used by the two groups, a new standard nomenclature for chicken β-defensins was proposed [36]. To be consistent with the mammalian defensin nomenclature, the term “gallinacin” was suggested to be dropped and a new term “avian β-defensin (AvBD)” be adopted [36]. The AvBD numbering system was proposed to follow Xiao’s [35]. With deposition of a new chicken β-defensin, *i.e.*, AvBD14 (under the GenBank accession no. AM402954), after initial two publications, the chicken genome now appears to encode a total of 14 distinct β-defensin genes (AvBD1–14) packed within a 85-kb distance toward one end of chromosome 3 [36]. In contrast with most mammalian β-defensin genes, which primarily consist of two exons, at least five AvBDs (AvBD1, 2, 6, 7, and 8) are comprised of a minimum of four exons [35]. The remaining AvBD genes contain three exons, while AvBD12 and -14 appear to have only two exons.

**Figure 3:**
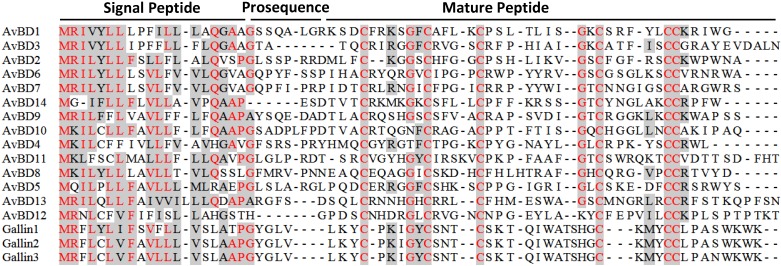
Amino acid sequence alignment of chicken β-defensins and ovodefensins. Conserved sequences are shaded and identical residues are in red. Dashes are created to maximize the alignment. Each β-defensin precursor is comprised of a conserved signal peptide, an optional short prosequence, and a C-terminal mature sequence consisting of six cysteines. Note that the cysteine spacing patterns are different between chicken ovodefensins (known as gallin 1–3) and classical β-defensins. Additional C-terminal tail sequences of AvBD3, 11, and 13 were omitted for simplicity.

Sequencing alignment of all 14 chicken β-defensins revealed that they are highly conserved at the N-terminal signal peptide region (Figure 3). The spacing pattern of six cysteines are also conserved at the C-terminal segment. Additionally, two glycines, with one preceding the second cysteine and the other preceding the fourth cysteine, are also largely conserved (Figure 3). Most other residues are quite diverse among AvBDs. In contrast to mammalian α-defensins with a long, often negatively charged prosequence, β-defensins including AvBDs consist of a short prosequence, which is even absent in a few cases. The C-terminal tails of AvBDs after the last cysteine are generally short, consisting mostly of 3-6 amino acids. AvBD3, 11 and 13 are exceptions. AvBD3 and AvBD13 have up to 30 residues after the last cysteine [35], while AvBD11 contains two tandem, but different six-cysteine motifs at the C-terminus [35]. As a result, mature AvBD11 may form six, instead of three, intramolecular disulfide bonds. AvBD11 is the only known β-defensin with such a sequence, and functional significance for the existence of such two defensin motifs remain to be studied. A number of AvBDs have also been found in several other species of birds including the turkey, ostrich, king penguin, zebra finch, duck, and goose [37,38,39,40,41,42,43]. Many additional AvBD sequences have also been predicted and deposited in GenBank.

Besides classical β-defensins, chickens have also been found to express three closely related β-defensin-related peptides, namely gallin 1–3 [23,44,45]. In fact, multiple peptides with a similar cysteine-spacing pattern have been reported earlier in the mallard duck, turkey, and black swan [46,47,48]. This group of peptides were in turn classified as ovodefensins because of their preferential expression in the oviduct with abundant presence in egg white [23]. Ovodefensins appear to be avian-specific, containing a six-cysteine motif of C-X_3-5_-C-X_3_-C-X_11_-C-X_3-4_-CC, as opposed to that of C-X_4-8_-C-X_3-5_-C-X_9-13_-C-X_4-7_-CC in classical avian β-defensins (Figure 3).

## 4. Evolution of Avian HDPs

### 4.1. Avian Cathelicidins

**Figure 4:**
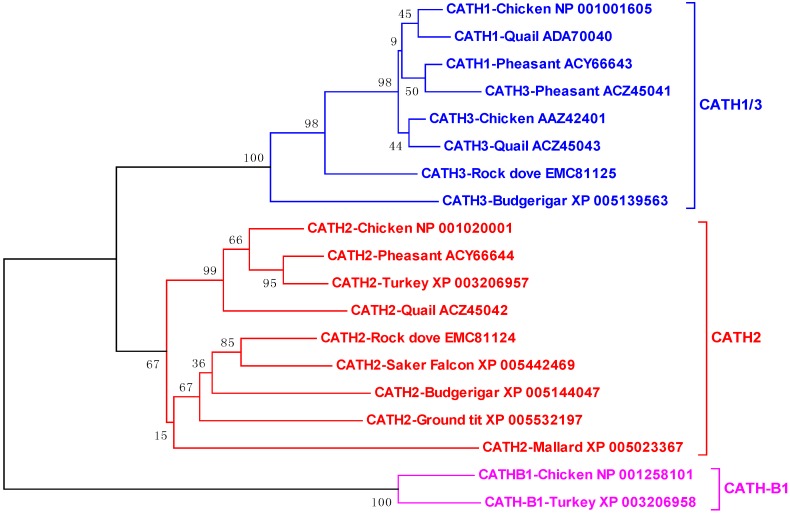
Phylogenetic analysis of avian cathelicidins. The phylogenetic tree was constructed with the full-length amino acid sequences using the neighbor-joining method, and the reliability of each branch was assessed by using 1,000 bootstrap replications. Numbers on the branches indicate the percentage of 1,000 bootstrap samples supporting the branch. The species and GenBank accession number of each sequence are indicated.

Phylogenetic analysis of all publically available avian cathelicidins revealed that they are clustered into three distinct clades, namely CATH1/3, CATH2, and CATH-B1 (Figure 4), suggesting that these three clades of cathelicidin genes have evolved before divergence of these bird species from each other, unlike mammalian cathelicidin genes, a majority of which were duplicated after species separation [18]. Surprisingly, an initial analysis of the zebra finch (*Taeniopygia guttata*) genome failed to identify any cathelicidin sequences [39]. If that is the case, it will be interesting to know why the cathelicidin genes were lost in zebra finch, although it is likely that the genome segments containing the cathelicidin genes failed to be sequenced, because the entire genome were only sequenced to 5.5 × coverage to encompass 94% of the genome [49]. Consistent with our assumption, scores of gaps are present in the syntenic cathelicidin region in the current zebra finch genome assembly (WUGSC 3.2.4/taeGut1) released in July 2008.

### 4.2. Avian β-Defensins

**Figure 5:**
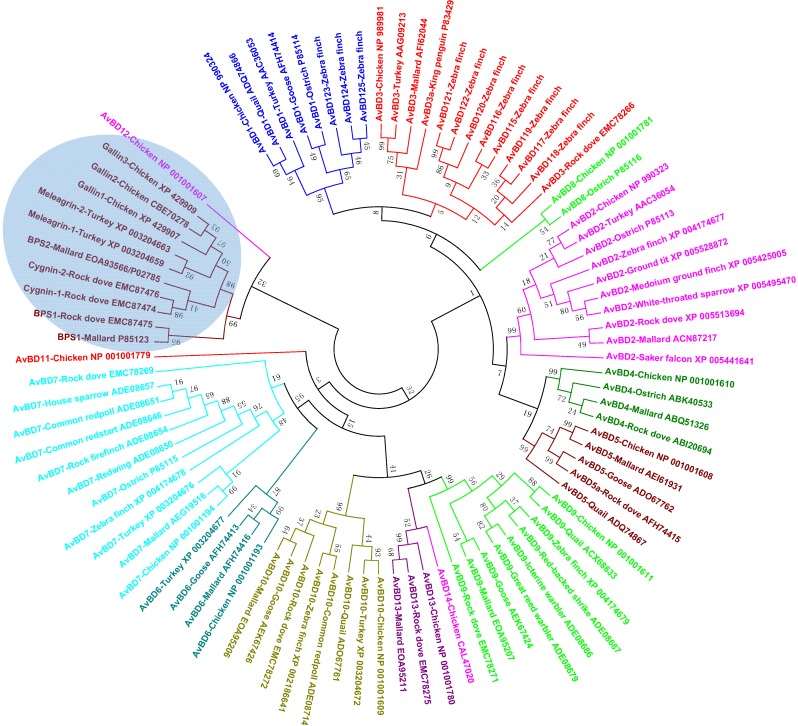
Phylogenetic analysis of avian defensins (see Figure 4 legend for details).

Phylogenetic analysis of all available avian β-defensins and related peptide sequences revealed the presence of cross-species, gene-specific clusters for most genes, implying that a majority have evolved before the split of the bird species from each other (Figure 5), reminiscent of avian cathelicidin genes. However, species-specific AvBD genes do exist. For example, AvBD14 appears specific to chickens, because no orthologs have been found in any other avian species, despite the availability of several avian genome sequences. Supported by a bootstrap value of 52 (Figure 5), it is likely that AvBD14 duplicated from AvBD13 after the separation of chickens from other birds. Zebra finch lacks the AvBD6 gene [39,40], although it is present in the chicken, turkey, goose, and mallard duck genomes. The AvBD1 gene is conserved in the chicken, turkey, goose, quail, and ostrich, but has apparently duplicated and diversified into three paralogous genes (AvBD123, 124, and 125) in the zebra finch [39,40] (Figure 5). Likewise, the AvBD3 gene has also expanded to a total of eight paralogous genes in the zebra finch genome [39,40] (Figure 5).

Ovodefensins clearly form a distinct clade, which is further divided into two subgroups (Figure 5). Small basic protein 1 (BPS1) in the mallard duck or rock dove is separated from the remaining ovodefensins. A closer examination of their amino acid sequences indicated that BPS1 consists of a cysteine-spacing pattern of C-X_3_-C-X_3_-C-X_11_-C-X_4_-CC, as opposed to C-X_5_-C-X_3_-C-X_11_-C-X_3_-CC in other ovodefensins. It remains to see whether these two subgroups of ovodefensins are present only in certain species of birds. It is also important to know how they are functionally different from each other and from classical defensins.

Importantly, ovodefensins are clustered with AvBD12 as supported by a bootstrap value of 32 (Figure 5), suggesting that ovodefensins might have duplicated and diversified from AvBD12 as a result of gene duplication after separations of birds from other animal species. Consistent with the notion that ovodefensins are derived from classical AvBDs, three chicken ovodefensin/gallin genes are located in tandem on chromosome 3, approximately 260 kb centromeric to the AvBD gene cluster on the current chicken genome assembly. To further support a possible origination of gallins from AvBD12, the gallin genes consist of two exons separated by an intron, which is identical to the genomic structure of the AvBD12 and AvBD14 genes, whereas all other AvBD genes are comprised of at least three exons [35].

## 5. Expression and Regulation of Avian HDPs

### 5.1. Tissue Expression Pattern

Like mammalian counterparts, avian cathelicidins and β-defensins are derived from the bone marrow and/or epithelial cells, with the majority expressing in a wide variety of tissues. CATH1–3 are primarily of myeloid origin, while CATH-B1, a distant member of avian cathelicidins, is derived from epithelial cells. Chicken CATH1–3 mRNAs are predominantly expressed in the bone marrow, but also throughout the mucosal tissues of the digestive, respiratory, and urogenital tracts [29,30,50]. On the other hand, chicken CATH-B1 mRNA shows a more restricted expression pattern, with preferential expression in the secretory epithelial cells of the bursa of Fabricius [31,50]. Consist with the role of cathelicidins in the first line of host defense, abundant CATH1–3 proteins can be detected in the granules of heterophils, as in the case of chicken CATH2 [51], whereas mature CATH-B1 protein is secreted from the epithelial cells and concentrated on the basolateral surfaces of the M cells in the bursal lymphoid follicles [31].

Myeloid avian β-defensins include AvBD1, 2, and 4–7, whereas the remaining AvBD8–14 are mainly of epithelial origin, although both myeloid and epithelial AvBDs are also expressed in a majority of other tissues [29,35,52]. In agreement with their myeloid origin, AvBD1 and AvBD2 mRNAs have been found abundantly in the bone marrow and their proteins in heterophil granules in the chickens, turkey, and ostrich [34,42,53]. It will be interesting to know the tissue expression pattern of those species-specific AvBDs such as AvBD115–125 in the zebra finch [40]. However, because they are orthologous to AvBD1 and AvBD3 (Figure 5), they are expected to share a similar expression pattern to AvBD1 and AvBD3.

Human cathelicidin LL-37 has been found in seminal plasma associated with sperm and prostasomes [54]. A majority of β-defensins in rats (and likely in other mammalian species as well) are expressed preferentially in the male reproductive system and the epididymis and testis in particular [55]. Like their mammalian counterparts, many avian cathelicidins and β-defensins are expressed adequately in the male and female reproductive organs, particularly in the testis, epididymis, ovary, and oviduct [29,35,50,56], suggesting a possible role in reproduction. Ovodefensins have been found to be among the major components of egg white in the chicken, turkey, and duck [44,45,46,47,48]. Consistently, chicken ovodefensin gallin 1–3 mRNA and proteins are the most abundantly expressed in tubular gland cells in the magnum of the chicken oviduct [23], a segment that secretes egg white.

### 5.2. Developmental Regulation

The expression of chicken cathelicidins and β-defensins has been studied during the pre- and post-hatch periods and found to be developmentally regulated. At the embryonic stage, most chicken cathelicidin and AvBD mRNAs were detected as early as embryonic day 3 (E3), except for CATH-B1 and AvBD11, which did not appear until day E9 [57]. All four cathelicidin mRNA expression was generally increased as embryos develops, whereas the 14 β-defensins were differentially expressed [57]. AvBD3, 4, 5, 10, 11, 12, and 14 were largely enhanced during the embryonic development, whereas the remaining chicken β-defensins showed a biphasic expression pattern. In the case of AvBD2, 6, and 7, their expression was increased on day E6 relative to that in day E3, decreased on day E9, and then increased gradually with the age of embryos [57].

After hatch, chicken cathelicidins and β-defensins are also developmentally regulated in both gene- and tissue-specific patterns. During the first 28 days, CATH1–3 showed an age-dependent increase both in the cecal tonsil and lung, whereas all four cathelicidins were peaked in the bursa on day 4 after hatching, with a gradual decline by day 28 [50]. On the other hand, CATH1–3 showed a peak expression in the cecum on day 28, while the highest expression of CATH-B1 was seen in both the lung and cecal tonsil on day 14 [50]. AvBD1 and AvBD2 mRNA gradually reduced in different segments of the intestinal tract in the first week post-hatch, but restored and increased gradually in the following week [58]. In the reproductive tract, more than a half of AvBDs increased during the sexual maturation in the vagina, ovary, and epididymis of chickens, whereas others showed little no expression [56,59,60].

### 5.3. Regulation by Infection and Inflammation

HDPs are critically involved in the first line of host defense. Dysregulation of the HDP synthesis often leads to immune deficiency or autoimmunity such as Crohn’s disease and psoriasis in humans [61,62]. Many, but not all, HDPs are induced upon infection and inflammation in humans and mice. On the other hand, certain pathogens suppress HDP synthesis as a strategy to evade the immune system [63,64,65]. A number of studies have been conducted on the transcriptional response of cathelicidins and β-defensins in chickens and several other avian species. Like their mammalian relatives, multiple AvBDs are inducible in response to microbial products (e.g., lipopolysaccharides and CpG DNA), live bacteria, viruses or parasites in the intestinal, reproductive, and respiratory tracts [38,56,59,60,66,67]. However, it appears that many avian HDP genes are regulated differently in different tissues in response to different stimuli. For example, chicken CATH1 was induced in the cecal tonsil [66], but not the jejunum of chickens in response to *Salmonella* infections [51]. On the other hand, chicken CATH1 was down-regulated by *Campylobacter jejuni* or *Eimeria praecox* [68,69,70], perhaps as a mechanism of immune evasion.

### 5.4. Regulation by Dietary Compounds

Because suppressing HDP expression is a microbial strategy for immune subversion, inducing the synthesis of endogenous HDPs will conversely augment the host capacity to fight off infections [71,72,73]. Unlike infection or injury that triggers HDP expression with an unwanted and often exaggerated inflammatory response, butyrate and vitamin D3 have been found to be highly potent in augmenting HDP synthesis without provoking inflammation in humans [74,75,76,77,78]. Several other compounds like dietary histone deacetylase inhibitors, retinoic acid, forskolin, and sugars are also capable of inducing HDP expression in humans [79,80,81,82,83].

In chickens, butyrate has been revealed as a strong inducer of HDP expression *in vitro* and *in vivo*. Among all 14 AvBDs and 4 cathelicidins, half of them were induced with others largely unchanged in chicken cells in response to butyrate [84]. Butyrate was further shown to enhance the antibacterial activity of chicken monocytes, and supplementation of butyrate in the feed enhanced clearance of *Salmonella enteritidis* in the cecum of chickens following an experimental infection [84]. Among all saturated free fatty acids with 1–18 carbons, butyrate was the most potent in stimulating HDP expression in chicken cells [85]. Furthermore, butyrate synergizes with the agonists of the cyclic adenosine monophosphate (cAMP) signaling pathway in inducing HDP expression [86]. Feeding with butyrate and a plant extract containing forskolin, which is an adenylyl cyclase agonist, showed a strong synergy in augmenting HDP expression in the crop and jejunum of chickens [86]. Mitogen-activated protein kinase signaling pathways were revealed to be critically involved in the HDP-inducing synergy between butyrate and forskolin [86]. The results indicated the potential for use of these dietary compounds in promoting HDP synthesis, host immunity, and disease resistance.

## 6. Biological Activities of Avian HDPs

### 6.1. Antimicrobial Activities

The antibacterial efficacy of all four chicken cathelicidins and many defensins have been evaluated. Like their mammalian counterparts, most chicken HDPs are capable of killing a broad spectrum of Gram-positive and Gram-negative bacteria, and fungi including antibiotic-resistant strains generally in the low micromolar range. For example, chicken CATH1–3 are broadly active with the minimum inhibitory concentration (MIC) values mostly between 0.5 and 2 µM against a range of bacteria [18,87], and CATH-B1 also has the MIC values between 0.5 and 2.5 µM against *E. coli, S. aureus*, and *P. aeruginosa* [31]. However, many HDPs showed varying efficiencies against different pathogens. Chicken AvBD1 and AvBD2 kill 90% *S. enteriditis*, *C. jejuni*, and *Candida albicans* at <4 µM, but showed a much reduced efficiency against *Pasteurella multocida* [41]. Similarly, AvBD9 is active against most Gram-positive and Gram-negative bacteria tested with the MIC values in the range of 2–4 µM, but with a minimum activity against *S. typhimurium* (>30 µM) [88]. On the other hand, AvBD13 was found to be minimally active against a range of bacteria examined, with the MIC values in the range of 50–100 µM [89]. The antibacterial activity of chicken CATH1–3 is not affected by the presence of physiological concentrations of salt [18]; however, that of chicken defensins is greatly reduced by salt [26], reminiscent of mammalian defensins [13]. In the case of CATH1–3, AvBD2, and presumably most other avian HDPs, non-specific membrane disruption and lysis is a major bactericidal mechanism [87,90,91].

Ovodefensins are unique in that they generally lack an obvious antibacterial activity as seen with turkey meleagrin as well as duck BPS1 and BPS2 [46,48]. Chicken gallin is the only ovodefensin with known antibacterial activities, but appears to have a narrow range. Among several common Gram-negative and Gram-positive bacteria tested, chicken gallin1/2 showed an activity only against *E. coli*, but not *Salmonella*, *S. aureus* or *Listeria monocytogenes* [24]. It is hence very unlikely that the major biological function of ovodefensins is antibacterial.

### 6.2. Immunomodulatory Activities

Besides having direct microbicidal activities, HDPs have increasingly been appreciated to play a profound role in regulating host immune responses to infections. Many peptides are shown to be actively involved in chemotaxis and activation of immune cells, regulation of dendritic cell differentiation, induction of angiogenesis and re-epithelialization, modulation of cytokine and chemokine gene expression, and potentiation of antigen-specific adaptive immune response [5,92,93]. Importantly, many HDPs directly bind to and neutralize bacterial membrane components such as lipopolysaccharides (LPS), lipotechoic acid, and peptidoglycan and suppress the production of proinflammatory cytokines induced by bacteria and membrane components [94,95].

In chickens, CATH1 was shown to possess excellent immunomodulatory properties with a strong capacity to specifically chemoattract neutrophils without affecting the migration of monocytes or lymphocytes [96]. Furthermore, CATH1 and CATH2 activates macrophages or peripheral blood mononuclear cells by inducing synthesis of an array of cytokines and chemokines at moderate levels, which is distinct from that induced by LPS [96,97], CATH1–3 were shown to bind to LPS directly, with 50% binding occurring at approximately 10 μM [18,87]. Moreover, three peptides at 10–20 μM substantially abrogated LPS-induced production of proinflammatory cytokines in macrophages and peripheral blood mononuclear cells [18,87,97]. CATH1 was further found to augment adaptive immune response when administered into mice together with chicken ovalbumin, a model antigen [96]. In the case of human cathelicidin LL-37, glyceraldehyde 3-phosphate dehydrogenase (GAPDH) and sequestosome-1/p62 were recently identified as intracellular receptors to mediate cytokine/chemokine production in monocytes [98,99]. It will be interesting to examine whether chicken cathelicidins also utilize the same receptors to modulate the macrophage response.

## 7. Structures-Activity Relationships of Avian HDPs

### 7.1. Structural Features

**Figure 6:**
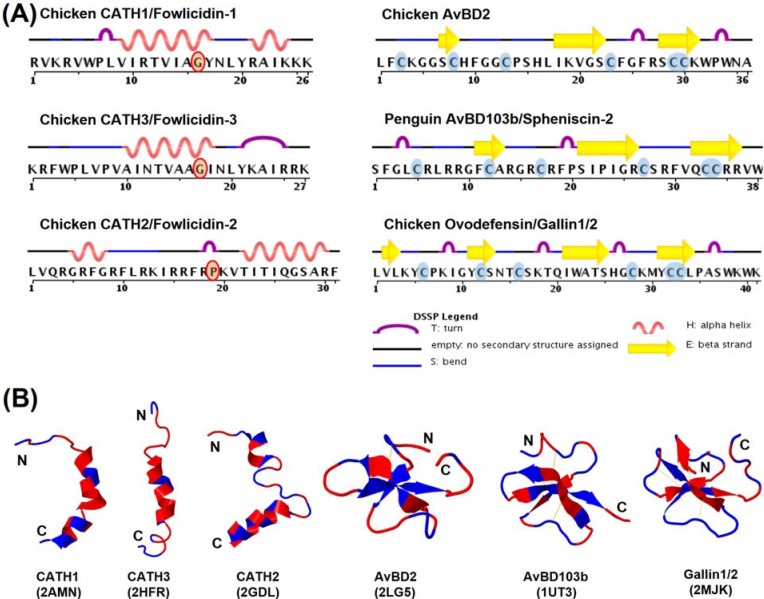
Structures of avian cathelicidins and β-defensins. (**A**) Secondary structural features of cathelicidins and β-defensins. (**B**) Tertiary ribbon structures of cathelicidins and β-defensins. Polar residues are indicated in blue and nonpolar residues in red. Disulfide bonds of β-defensins are shown in yellow. Protein Data Bank identification number for each molecule is indicated in parenthesis.

To date, the tertiary structures of three chicken cathelicidins (CATH1–3), two β-defensins (chicken AvBD2 and penguin AvBD103a/spheniscin-2), and a chicken ovodefensin (gallin1/2) have been determined by nuclear magnetic resonance (NMR) in solutions. Unlike mammalian cathelicidins that adopt various conformations, all three chicken cathelicidins are largely α-helical with a mild kink or rather extensive bend around the center in aqueous solutions [87,90,100] (Figure 6). A mild kink in CATH1 and CATH3 is induced by the presence of a glycine residue, whereas an extensive bend in CATH2 is caused by proline (Figure 6A). Additionally, all three chicken cathelicidins consist of a flexible unstructured segment at the N-terminal region [87,90,100]. Unlike typical amphipathic α-helical HDPs, no obvious segregation of hydrophobic residues from hydrophilic residues is seen with either CATH1 or CATH2; instead, the positively charged residues are mostly concentrated at both ends (Figure 6B). On the other hand, CATH2 are rather amphipathic throughout the entire α-helix. Three truncated analogs of chicken CATH1 consisting of amino acid residues 1–16, 8–26, and 5–26 were also revealed to adopt similar confirmations to the full-length peptide in the presence of LPS or zwitterionic dodecylphosphocholine micelles [101,102].

Both penguin AvBD103a and chicken AvBD2 adopt a triple-stranded, antiparallel β-sheet structure stabilized by three pairs of intramolecular disulfide bonds [91,103] (Figure 6B), typical of mammalian β-defensins. Penguin AvBD103a also consists of an α-helical segment at the N-terminal region with a hydrophobic patch on the surface [103]. However, AvBD2 lacks either the α-helical or an obvious amphipathic feature [91]. A β-bulge that is formed by the G-X-C motif around the fourth cysteine and highly conserved in both mammalian α- and β-defensins is also present in both AvBD2 and AvBD103a (Figure 6B). Albeit with a different cysteine-spacing pattern, chicken gallin1/2 also consists of a characteristic β-defensin fold with three antiparallel β-sheets [24]. However, unlike classical β-defensins, gallin1/2 is comprised of two additional β-sheets formed separately by Val^2^-Leu^3^ at the N-terminal end and Thr^24^-Ser^25^ preceding the fourth cysteine [24] (Figure 6).

### 7.2. Structure-Activity Relationships

Structure-activity relationship (SAR) studies with α-helical HDPs indicated that the antimicrobial potency and target specificity are strongly influenced by structural and physicochemical parameters, such as cationicity (net charge), helicity, amphipathicity, and hydrophobicity [11,104,105]. However, in general there is no simple correlation between any of these physicochemical properties and peptide functions. A delicate balance of these parameters often dictates the antimicrobial potency and target selectivity [11,104,105]. Within a certain range, an improvement in these parameters is often positively correlated with the antimicrobial activity of peptides, but sometimes is accompanied by unwanted enhancement in cytotoxicity as well [104,105,106,107]. In several cases, the antimicrobial domain of the peptides is located separately from the domain responsible for cytotoxicity [108,109], meaning that the peptide derivatives devoid of the lytic domain could be identified with improved therapeutic potential.

In the case of β-sheet HDPs and defensins in particular, antimicrobial and immunomodulatory activities are strongly influenced by structural integrity, cationicity, and hydrophobicity [11,110,111]. The presence of three intramolecular disulfide bonds in many cases is dispensable for the antibacterial activity, but essential for other activities such as chemotactic activity [112] and the ability to resist proteolysis [113]. On the contrary, the antibacterial activity of human β-defensin-1 drastically increases when the peptide is reduced [114]. In fact, some defensins may be naturally reduced in the intestinal tract by thioredoxin, a redox enzyme [114]. Cationicity of defensins is believed to dictate the killing of Gram-negative bacteria, whereas hydrophobicity appears to confer the activity against Gram-positive bacteria [111].

In avian species, a series of SAR studies with chicken CATH1, CATH2, and AvBD2 have yielded some very interesting observations. Investigations of chicken CATH1 analogs with either N- or C-terminal deletions revealed that the cationic residues at both N- and C-terminal regions are dispensable for the antibacterial, LPS-binding, and cytotoxic activities, whereas the C-terminal helix (Arg^21^-Lys^25^) is essential for all three activities [100]. Tryptophan at position 6 (Trp^6^) is critical in both LPS binding and cytotoxicity [100,115], but is dispensable for neutrophil chemotaxis [96]. Furthermore, an omission of Trp^6^ in CATH1 also resulted in an obvious reduction in its ability to kill bacteria [115] and induce chemokine synthesis in macrophages [96]. Replacing the kink-causing glycine (Gly^16^) with a helix-stabilizing residue, leucine, resulted in no obvious difference in either antibacterial, LPS-binding or cytotoxic activity [100], indicating that enhancing the helicity of α-helical HDPs may not necessarily result in an improvement in the antibacterial potency. Simultaneous substitutions of multiple amino acid residues to make CATH1 nearly perfectly amphipathic surprisingly caused a loss of the antibacterial potency against certain bacteria and undesirably, an increase in hemolytic activities [100]. Collectively, these findings suggested that a fine tuning of various structural and physicochemical parameters including cationicity, helicity, hydrophobicity, and amphipathicity, rather than a simple alteration of one, will result in an enhancement in the therapeutic potential of α-helical HDPs, which is in agreement with earlier findings [11,104,105].

Studies with a series of CATH2 analogs with deletions of either N- or C-terminal residues showed that neither α-helical segment *per se* is sufficient to bind LPS, kill bacteria or lyse mammalian cells [90]. Inclusion of four additional amino acids in the central bending region (Arg^15^-Arg^18^) beyond either N- or C-terminal α-helical segment to the α-helical segment was associated with a significant enhancement in both antibacterial and LPS-neutralizing activities [90]. To directly evaluate the functional significance of the central bending segment, a substitution of leucine for proline greatly reduced the antibacterial and hemolytic activities. The abilities to neutralize LPS-induced cytokine production and to stimulate chemokine synthesis in peripheral blood mononuclear cells were also significantly impaired by such a proline-to-leucine substitution [97], implying that the bending is critically important in the peptide interactions with membranes as well as the cell activation receptors. Interestingly, a gradual increase in cationicity, helicity and amphipathicity among all peptide analogs led to a gradual enhancement in antibacterial potency and LPS neutralization [90]. Furthermore, substitution of multiple tryptophans for phenylalanines in an N-terminal, 15-amino acid fragment of CATH2 led to an improvement in antibacterial and LPS-neutralizing activities [116]. Head-to-tail cyclization of this CATH2 variant further increased its serum stability with a reduced cytotoxicity [117]. The same study also revealed that d-amino acid substitutions rendered the peptide completely resistant to trypsin proteolysis [117].

In the case of chicken AvBD2, d-enantiomerization resulted in little difference in the activity against both Gram-positive and Gram-negative bacteria, suggesting that membrane is the primary target [91]. However, unlike many mammalian defensins whose structural integrity has a minimum impact on the antibacterial activity, reducing AvBD2 led to a drastic loss in the activity against Gram-positive bacteria, but was less prominent against Gram-negative bacteria [91]. Substitution of alanine for a conserved lysine following the sixth cysteine resulted in an obvious N-terminal structural modification and a marked decrease in the antibacterial activity against both Gram-positive and Gram-negative bacteria [91]. Surprisingly, a reduction of disulfide bonds rendered the lysine-alanine substituted AvBD2 nearly completely inactive in killing bacteria [91]. Therefore, conformational changes (and subsequent changes in hydrophobicity and/or amphipathicity) are likely the underlying mechanism behind many of the alterations in the antibacterial activity of AvBD2.

## 8. Potential Therapeutic Applications

**Figure 7:**
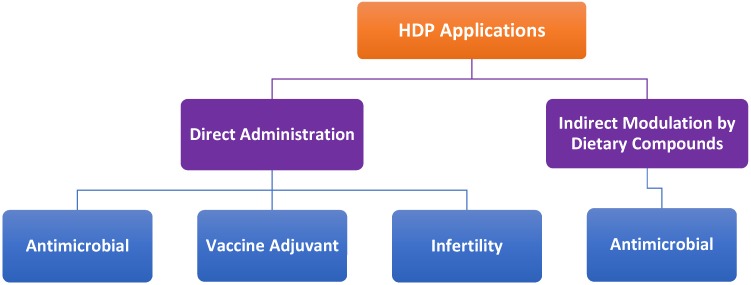
Potential therapeutic applications of host defense peptides (HDPs). Synthetic HDPs can be directly administered exogenously as antimicrobials, vaccine adjuvants or infertility drugs. Alternatively, endogenous HDPs can modulated by dietary compounds for antimicrobial therapies.

HDPs including avian HDPs can be potentially used in a variety of applications such as antimicrobial therapy (Figure 7). Additionally, HDPs hold promise in augmenting the efficacy of vaccines as adjuvants. Although a number of avian HDPs are expressed in both male and female reproductive tracts and believed to promote sperm maturation and fertility like their mammalian counterparts [10,118], experimental evidence is yet to prove the link. Therefore, the potential role and application of avian HDPs in infertility treatment will not be discussed here. To reduce the cost of delivering synthetic peptides and minimize peptide degradation, endogenous HDPs can also be induced by certain cost-effective dietary compounds to help the host better fight off infections (Figure 7).

### 8.1. Antimicrobial Therapies

HDPs are active against a broad range of bacteria, mycobacteria, fungi, and parasites [1,8]. Rather than relying on a single or a limited number of intracellular targets like most currently available antibiotics, HDPs kills microbes primarily through physical electrostatic interactions and membrane disruption. Therefore, it is difficult for microbes to gain resistance to HDPs [1,8]. At the same time, most HDPs have the capacity to recruit and activate immune cells and facilitate the resolution of inflammation [6,8]. In fact, the antibacterial and immunomodulatory properties of HDPs can be harnessed separately for antimicrobial therapy, particularly against antibiotic-resistant strains [6,8]. A few HDPs have been evaluated clinically for their antibacterial efficacy, and several more are currently at different stages of human trials. Because of a relatively low efficacy as compared with many of the conventional antibiotics, all clinical trials with HDPs have met a limited success [119]. As a result, no HDPs have been approved by the FDA to date. More efforts are being shifted toward exploring the immune regulatory activities of HDPs. Excitedly, several small HDPs with no or weak antibacterial activities have been proved to be highly efficient in protecting animals from infections by recruiting and activating neutrophils and/or monocytes [95,120]. Because they act on the host but not on the pathogens, these immunomodulatory peptides have the potential to control a broad spectrum of pathogens without triggering resistance.

In avian species, only chicken CATH1 has been evaluated for its *in vivo* antibacterial efficacy. A single intraperitoneal administration of a C-terminal, 21-amino acid CATH1 peptide analog, known as fowlicidin-1 (6–26), led to a 50% increase in the survival of mice from a lethal dose of methicillin-resistant *S. aureus* (MRSA), concomitant with a reduction in the bacterial titer in both peritoneal fluids and spleens of mice [115]. Additionally, fowlicidin-1(6–26) is more potent in inducing neutrophil chemotaxis and macrophage activation than human cathelicidin LL-37 and a *de novo* synthesized peptide, IDR-1 [96]. Because of its ability to induce neutrophil chemotaxis and macrophage activation, fowlicidin-1(6–26) protected 50% mice if given 4 days prior to an otherwise lethal MRSA infection, and 100% mice survived if the peptide was received 1 or 2 days before infection [96]. This is the first HDP that has been shown to protect animals from bacterial infections beyond a 48-h window. Therefore, fowlicidin-1(6–26) represents an attractive candidate for further exploration as a novel antimicrobial for both therapeutic and prophylactic applications. Rational design and functional screening of additional CATH1-related peptides may lead to identification of new peptide analogs with improved safety and therapeutic potential, particularly against antibiotic-resistant pathogens.

It is worth noting that many HDPs and defensins in particular have obvious antiviral effects by acting as lectins or by modulating host cell responses. Enveloped and non-enveloped viruses such as human immunodeficiency virus (HIV-1), influenza A virus (IAV), cytomegalovirus (CMV), herpes simplex virus (HSV-1 and HSV-2), vesicular stomatitis virus (VSV) , adenovirus, and papillomavirus (HPV) have been shown to be sensitive to human α-, β- and/or θ-defensins [121,122]. In some cases, defensins can directly inactivate viruses by disrupting envelop lipid bilayers, aggregating viral glycoproteins or blocking the binding of viruses to host cell receptors [122]. In other cases, the antiviral effects of defensins are indirectly mediated by modifying host cell responses such as inhibition of protein kinase C (PKC) activation or down-regulation of host cell receptor expression [122]. Limited work exists on the antiviral activities of avian HDPs. Only a few duck β-defensins were recently shown to inhibit the replication of duck hepatitis virus (DHV) [37,67]. However, the antiviral mechanisms or the susceptibility of other viruses to avian HDPs remains unknown, but warrant further investigations.

### 8.2. Vaccine Adjuvants

HDPs have been shown to profoundly impact the development of adaptive immune response by regulating the migration, maturation, and activation of different immune cell types including dendritic cells and T and B lymphocytes [5,8,123]. Several HDPs are capable of enhancing antigen-specific adaptive immune response when co-administered with vaccines [123]. HDPs have been found to synergize with other adjuvants like CpG DNA and polyphosphazene in potentiating adaptive immune response [124,125,126]. The adjunvanticity of chicken AvBD1, duck AvBD2, and chicken CATH1 have been experimentally verified. When fused with the infectious bursal disease virus (IBDV) VP2 gene in a DNA vaccine, chicken AvBD1 increased the VP2-specific antibody titers, CD4^+^ and CD8^+^ T cell populations, and conferred better protection against an infectious bursal disease virus (IBDV) challenge in chickens [127]. Duck AvBD2 was shown to be chemotactic to T- and B-lymphocytes *in vitro*, with the ability to suppress the mRNA expression of an inhibitory receptor, namely dendritic cell immunoreceptor (DCIR), in duck splenocytes [128]. Chicken CATH1, when co-administered with chicken ovalbumin (OVA), was found to enhance both IgG_1_ and IgG_2a_ titers to OVA in mice [96]. Because CATH 1 was more potent than LL-37 or IDR-1 in inducing surface expression of CD86, a co-stimulatory molecule, on macrophages [96], CATH1 may be more efficient in promoting antigen presentation and adaptive immunity and therefore, represent an excellent candidate as an adjuvant or a component of an adjuvant complex.

### 8.3. Direct Administration *vs.* Indirect Modulation

The production cost and stability are two major obstacles for *in vivo* applications of many peptide-based drugs. Purification from natural sources or chemical synthesis are inefficient and cost-prohibitive for large-scale production of HDPs. Although recombinant expression of HDPs has been achieved in bacteria and yeasts, an inclusion of a fusion protein is often needed to reduce the peptide toxicity to the host and aid in the peptide solubility [129]. In order to achieve the maximum activity, an extra proteolytic cleavage step and additional production costs are often unavoidable. Given a short half-life of most natural peptides *in vivo*, it is desirable to retard the peptide degradation by chemical modifications that often involves the use of d-amino acids, cyclization or peptidomimetics [130]. However, it remains unknown how those modifications would impact the immunomodulatory functions of HDPs, which appear to be receptor-dependent.

To overcome the high manufacturing cost and minimize degradation of HDPs for *in vivo* applications, it is advantageous to develop convenient and cost-effective strategies to specifically induce the synthesis of endogenous HDPs. Several dietary compounds including short-chain fatty acids and vitamin D_3_ have shown promise in stimulating HDP synthesis in humans without triggering inflammatory response [131,132]. Dietary supplementation of HDP-inducing compounds has emerged as a novel antibiotic-alternative approach to antimicrobial therapy [131,132]. In chickens, butyrate, structural analogs of butyrate, and cAMP signaling agonists have been shown to be potent inducers of HDPs [84,85,86]. Desirably, butyrate and cAMP agonists are synergistic in augmenting HDP gene expression and bacterial clearance in chickens [86], suggesting their potential as alternatives to antibiotics for disease control and prevention. However, dietary regulation of HDPs often exhibit gene-, cell-, and species-specific patterns. HDP genes are differentially regulated in response to a dietary compound, with some being induced and others unaltered. In chickens, approximately a half number of chicken HDPs are induced by butyrate [84]. Some HDPs are regulated in a cell-specific pattern. For example, chicken AvBD9 gene increased by more than 5000-fold in HD11 macrophages, but with only a less than 10-fold induction in cecal intestinal cells after a 24-h exposure to 4 mM butyrate [84]. The same compound that show a strong HDP-inducing activity in one animal species, may completely lose its ability to induce HDPs in another species. A case in point is vitamin D3, which strongly augments cathelicidin gene expression in human but not mouse cells [75,76]. Therefore, it is important to evaluate the HDP-inducing efficacy of individual compounds in different species.

## 9. Conclusions

Birds harbor approximately 20 unique cathelicidins and β-defensin genes in each species. It appears that most have evolved before divergence of birds from each other. Unlike mammalian cathelicidins that adopt different tertiary structures, avian cathelicidins are mostly α-helical, with a hinge around the central region and a flexible N-terminal segment. Besides classical β-defensins, a group of avian-specific, β-defensin-related peptides, namely ovodefensins, exist with a different cysteine-spacing pattern. However, the overall three-dimensional structure of ovodenfensins resemble that of β-defensins. Coupled with their close chromosomal proximity with the β-defensin gene cluster, ovodefensins were clearly diversified from a β-defensin ancestral gene, possibly AvBD12, after separation of the birds from other vertebrate species. Several avian HDPs have been shown to possess potent, broad-spectrum antibacterial activities with a strong ability to modulate the host response to infection and inflammation. Structure-activity relationship studies have led to the identification of a few avian HDPs and their analogs as promising candidates as antimicrobials or vaccine adjuvants. Because avian HDPs can be induced by dietary compounds such as short-chain fatty acids and cAMP signaling agonists, dietary modulation of endogenous HDP synthesis may have potential to be further explored as a novel, cost-effective antimicrobial strategy.

## References

[1] Zasloff M. (2002). Antimicrobial peptides of multicellular organisms. Nature.

[2] Pasupuleti M., Schmidtchen A., Malmsten M. (2012). Antimicrobial peptides: Key components of the innate immune system. Crit. Rev. Biotechnol..

[3] Wang G. (2013). Database-Guided Discovery of Potent Peptides to Combat HIV-1 or Superbugs. Pharmaceuticals (Basel).

[4] Brogden K.A. (2005). Antimicrobial peptides: Pore formers or metabolic inhibitors in bacteria?. Nat. Rev. Microbiol..

[5] Yang D., Biragyn A., Hoover D.M., Lubkowski J., Oppenheim J.J. (2004). Multiple roles of antimicrobial defensins, cathelicidins, and eosinophil-derived neurotoxin in host defense. Annu. Rev. Immunol..

[6] Choi K.Y., Chow L.N., Mookherjee N. (2012). Cationic host defence peptides: Multifaceted role in immune modulation and inflammation. J. Innate Immun..

[7] Hilchie A.L., Wuerth K., Hancock R.E. (2013). Immune modulation by multifaceted cationic host defense (antimicrobial) peptides. Nat. Chem. Biol..

[8] Hancock R.E., Nijnik A., Philpott D.J. (2012). Modulating immunity as a therapy for bacterial infections. Nat. Rev. Microbiol..

[9] Semple F., Dorin J.R. (2012). β-Defensins: Multifunctional modulators of infection, inflammation and more?. J. Innate Immun..

[10] Tollner T.L., Bevins C.L., Cherr G.N. (2012). Multifunctional glycoprotein DEFB126—A curious story of defensin-clad spermatozoa. Nat. Rev. Urol..

[11] Takahashi D., Shukla S.K., Prakash O., Zhang G. (2010). Structural determinants of host defense peptides for antimicrobial activity and target cell selectivity. Biochimie.

[12] Zanetti M. (2004). Cathelicidins, multifunctional peptides of the innate immunity. J. Leukoc. Biol..

[13] Selsted M.E., Ouellette A.J. (2005). Mammalian defensins in the antimicrobial immune response. Nat. Immunol..

[14] Ganz T. (2003). Defensins: Antimicrobial peptides of innate immunity. Nat. Rev. Immunol..

[15] Kosciuczuk E.M., Lisowski P., Jarczak J., Strzalkowska N., Jozwik A., Horbanczuk J., Krzyzewski J., Zwierzchowski L., Bagnicka E. (2012). Cathelicidins: Family of antimicrobial peptides. A review. Mol. Biol Rep..

[16] Levy O., Ooi C.E., Elsbach P., Doerfler M.E., Lehrer R.I., Weiss J. (1995). Antibacterial proteins of granulocytes differ in interaction with endotoxin. Comparison of bactericidal/permeability-increasing protein, p15s, and defensins. J. Immunol..

[17] Moscinski L.C., Hill B. (1995). Molecular cloning of a novel myeloid granule protein. J. Cell. Biochem..

[18] Xiao Y., Cai Y., Bommineni Y.R., Fernando S.C., Prakash O., Gilliland S.E., Zhang G. (2006). Identification and functional characterization of three chicken cathelicidins with potent antimicrobial activity. J. Biol Chem..

[19] Zarember K.A., Katz S.S., Tack B.F., Doukhan L., Weiss J., Elsbach P. (2002). Host defense functions of proteolytically processed and parent (unprocessed) cathelicidins of rabbit granulocytes. Infect. Immun..

[20] Lehrer R.I. (2004). Primate defensins. Nat. Rev. Microbiol..

[21] Andersson M.L., Karlsson-Sjoberg J.M., Putsep K.L. (2012). CRS-peptides: Unique defense peptides of mouse Paneth cells. Mucosal Immunol..

[22] Patil A.A., Ouellette A.J., Lu W., Zhang G. (2013). Rattusin, an intestinal alpha-defensin-related peptide in rats with a unique cysteine spacing pattern and salt-insensitive antibacterial activities. Antimicrob. Agents Chemother..

[23] Gong D., Wilson P.W., Bain M.M., McDade K., Kalina J., Herve-Grepinet V., Nys Y., Dunn I.C. (2010). Gallin; an antimicrobial peptide member of a new avian defensin family, the ovodefensins, has been subject to recent gene duplication. BMC Immunol..

[24] Herve V., Meudal H., Labas V., Rehault Godbert S., Gautron J., Berges M., Guyot N., Delmas A.F., Nys Y., Landon C. (2014). 3D NMR structure of hen egg gallin (chicken ovo-defensin) reveals a new variation of the beta-defensin fold. J. Biol. Chem..

[25] Van Dijk A., Molhoek E.M., Bikker F.J., Yu P.L., Veldhuizen E.J., Haagsman H.P. (2011). Avian cathelicidins: Paradigms for the development of anti-infectives. Vet. Microbiol..

[26] Van Dijk A., Veldhuizen E.J., Haagsman H.P. (2008). Avian defensins. Vet. Immunol Immunopathol..

[27] Sugiarto H., Yu P.L. (2004). Avian antimicrobial peptides: The defense role of beta-defensins. Biochem. Biophys. Res. Commun..

[28] Cuperus T., Coorens M., van Dijk A., Haagsman H.P. (2013). Avian host defense peptides. Dev. Comp. Immunol..

[29] Lynn D.J., Higgs R., Gaines S., Tierney J., James T., Lloyd A.T., Fares M.A., Mulcahy G., O’Farrelly C. (2004). Bioinformatic discovery and initial characterisation of nine novel antimicrobial peptide genes in the chicken. Immunogenetics.

[30] Van Dijk A., Veldhuizen E.J., van Asten A.J., Haagsman H.P. (2005). CMAP27, a novel chicken cathelicidin-like antimicrobial protein. Vet. Immunol. Immunopathol..

[31] Goitsuka R., Chen C.L., Benyon L., Asano Y., Kitamura D., Cooper M.D. (2007). Chicken cathelicidin-B1, an antimicrobial guardian at the mucosal M cell gateway. Proc. Natl. Acad. Sci. USA.

[32] Feng F., Chen C., Zhu W., He W., Guang H., Li Z., Wang D., Liu J., Chen M., Wang Y., Yu H. (2011). Gene cloning, expression and characterization of avian cathelicidin orthologs, Cc-CATHs, from Coturnix coturnix. FEBS J..

[33] Wang Y., Lu Z., Feng F., Zhu W., Guang H., Liu J., He W., Chi L., Li Z., Yu H. (2011). Molecular cloning and characterization of novel cathelicidin-derived myeloid antimicrobial peptide from Phasianus colchicus. Dev. Comp. Immunol..

[34] Evans E.W., Beach G.G., Wunderlich J., Harmon B.G. (1994). Isolation of antimicrobial peptides from avian heterophils. J. Leukoc. Biol..

[35] Xiao Y., Hughes A.L., Ando J., Matsuda Y., Cheng J.F., Skinner-Noble D., Zhang G. (2004). A genome-wide screen identifies a single beta-defensin gene cluster in the chicken: Implications for the origin and evolution of mammalian defensins. BMC Genomics.

[36] Lynn D.J., Higgs R., Lloyd A.T., O'Farrelly C., Herve-Grepinet V., Nys Y., Brinkman F.S., Yu P.L., Soulier A., Kaiser P. (2007). Avian beta-defensin nomenclature: A community proposed update. Immunol. Lett..

[37] Ma D., Zhang K., Zhang M., Xin S., Liu X., Han Z., Shao Y., Liu S. (2012). Identification, expression and activity analyses of five novel duck beta-defensins. PLoS One.

[38] Ma D., Zhang M., Zhang K., Liu X., Han Z., Shao Y., Liu S. (2013). Identification of three novel avian beta-defensins from goose and their significance in the pathogenesis of Salmonella. Mol. Immunol..

[39] Cormican P., Lloyd A.T., Downing T., Connell S.J., Bradley D., O’Farrelly C. (2009). The avian Toll-Like receptor pathway—Subtle differences amidst general conformity. Dev. Comp. Immunol..

[40] Hellgren O., Ekblom R. (2010). Evolution of a cluster of innate immune genes (beta-defensins) along the ancestral lines of chicken and zebra finch. Immunome Res..

[41] Evans E.W., Beach F.G., Moore K.M., Jackwood M.W., Glisson J.R., Harmon B.G. (1995). Antimicrobial activity of chicken and turkey heterophil peptides CHP1, CHP2, THP1, and THP3. Vet. Microbiol..

[42] Sugiarto H., Yu P.L. (2006). Identification of three novel ostricacins: An update on the phylogenetic perspective of beta-defensins. Int. J. Antimicrob. Agents.

[43] Thouzeau C., Le Maho Y., Froget G., Sabatier L., Le Bohec C., Hoffmann J.A., Bulet P. (2003). Spheniscins, avian beta-defensins in preserved stomach contents of the king penguin, Aptenodytes patagonicus. J. Biol Chem..

[44] Mann K. (2007). The chicken egg white proteome. Proteomics.

[45] Mann K., Mann M. (2011). In-depth analysis of the chicken egg white proteome using an LTQ Orbitrap Velos. Proteome Sci.

[46] Odani S., Koide T., Ono T., Takahashi Y., Suzuki J. (1989). Covalent structure of a low-molecular-mass protein, meleagrin, present in a turkey (Meleagris gallopavo) ovomucoid preparation. J. Biochem..

[47] Simpson R.J., Morgan F.J. (1983). Isolation and complete amino acid sequence of a basic low molecular weight protein from black swan egg white. Int. J. Pept. Protein Res..

[48] Naknukool S., Hayakawa S., Ogawa M. (2011). Multiple biological functions of novel basic proteins isolated from duck egg white: Duck basic protein small 1 (dBPS1) and 2 (dBPS2). J. Agric. Food Chem..

[49] Warren W.C., Clayton D.F., Ellegren H., Arnold A.P., Hillier L.W., Kunstner A., Searle S., White S., Vilella A.J., Fairley S. (2010). The genome of a songbird. Nature.

[50] Achanta M., Sunkara L.T., Dai G., Bommineni Y.R., Jiang W., Zhang G. (2012). Tissue expression and developmental regulation of chicken cathelicidin antimicrobial peptides. J. Anim. Sci. Biotechnol..

[51] Van Dijk A., Tersteeg-Zijderveld M.H., Tjeerdsma-van Bokhoven J.L., Jansman A.J., Veldhuizen E.J., Haagsman H.P. (2009). Chicken heterophils are recruited to the site of Salmonella infection and release antibacterial mature Cathelicidin-2 upon stimulation with LPS. Mol. Immunol..

[52] Zhao C., Nguyen T., Liu L., Sacco R.E., Brogden K.A., Lehrer R.I. (2001). Gallinacin-3, an inducible epithelial beta-defensin in the chicken. Infect. Immun..

[53] Harwig S.S., Swiderek K.M., Kokryakov V.N., Tan L., Lee T.D., Panyutich E.A., Aleshina G.M., Shamova O.V., Lehrer R.I. (1994). Gallinacins: Cysteine-rich antimicrobial peptides of chicken leukocytes. FEBS Lett..

[54] Andersson E., Sorensen O.E., Frohm B., Borregaard N., Egesten A., Malm J. (2002). Isolation of human cationic antimicrobial protein-18 from seminal plasma and its association with prostasomes. Hum. Reprod..

[55] Patil A.A., Cai Y., Sang Y., Blecha F., Zhang G. (2005). Cross-species analysis of the mammalian beta-defensin gene family: Presence of syntenic gene clusters and preferential expression in the male reproductive tract. Physiol. Genomics.

[56] Anastasiadou M., Avdi M., Michailidis G. (2013). Expression of avian beta-defensins and Toll-like receptor genes in the rooster epididymis during growth and Salmonella infection. Anim. Reprod. Sci..

[57] Meade K.G., Higgs R., Lloyd A.T., Giles S., O’Farrelly C. (2009). Differential antimicrobial peptide gene expression patterns during early chicken embryological development. Dev. Comp. Immunol..

[58] Bar-Shira E., Friedman A. (2006). Development and adaptations of innate immunity in the gastrointestinal tract of the newly hatched chick. Dev. Comp. Immunol..

[59] Anastasiadou M., Avdi M., Theodoridis A., Michailidis G. (2013). Temporal changes in the expression of avian beta-defensins in the chicken vagina during sexual maturation and Salmonella infection. Vet. Res. Commun..

[60] Michailidis G., Avdi M., Argiriou A. (2012). Transcriptional profiling of antimicrobial peptides avian beta-defensins in the chicken ovary during sexual maturation and in response to Salmonella enteritidis infection. Res. Vet. Sci..

[61] Gersemann M., Wehkamp J., Stange E.F. (2012). Innate immune dysfunction in inflammatory bowel disease. J. Intern. Med..

[62] Gilliet M., Lande R. (2008). Antimicrobial peptides and self-DNA in autoimmune skin inflammation. Curr. Opin. Immunol..

[63] Islam D., Bandholtz L., Nilsson J., Wigzell H., Christensson B., Agerberth B., Gudmundsson G. (2001). Downregulation of bactericidal peptides in enteric infections: A novel immune escape mechanism with bacterial DNA as a potential regulator. Nat. Med..

[64] Bergman P., Johansson L., Asp V., Plant L., Gudmundsson G.H., Jonsson A.B., Agerberth B. (2005). Neisseria gonorrhoeae downregulates expression of the human antimicrobial peptide LL-37. Cell. Microbiol..

[65] Chakraborty K., Ghosh S., Koley H., Mukhopadhyay A.K., Ramamurthy T., Saha D.R., Mukhopadhyay D., Roychowdhury S., Hamabata T., Takeda Y. (2008). Bacterial exotoxins downregulate cathelicidin (hCAP-18/LL-37) and human beta-defensin 1 (HBD-1) expression in the intestinal epithelial cells. Cell. Microbiol..

[66] Akbari M.R., Haghighi H.R., Chambers J.R., Brisbin J., Read L.R., Sharif S. (2008). Expression of antimicrobial peptides in cecal tonsils of chickens treated with probiotics and infected with Salmonella enterica serovar typhimurium. Clin. Vaccine Immunol..

[67] Ma D., Lin L., Zhang K., Han Z., Shao Y., Liu X., Liu S. (2011). Three novel Anas platyrhynchos avian beta-defensins, upregulated by duck hepatitis virus, with antibacterial and antiviral activities. Mol. Immunol..

[68] Meade K.G., Narciandi F., Cahalane S., Reiman C., Allan B., O’Farrelly C. (2009). Comparative *in vivo* infection models yield insights on early host immune response to Campylobacter in chickens. Immunogenetics.

[69] Van Dijk A., Herrebout M., Tersteeg-Zijderveld M.H., Tjeerdsma-van Bokhoven J.L., Bleumink-Pluym N., Jansman A.J., Veldhuizen E.J., Haagsman H.P. (2012). Campylobacter jejuni is highly susceptible to killing by chicken host defense peptide cathelicidin-2 and suppresses intestinal cathelicidin-2 expression in young broilers. Vet. Microbiol..

[70] Sumners L.H., Miska K.B., Jenkins M.C., Fetterer R.H., Cox C.M., Kim S., Dalloul R.A. (2011). Expression of Toll-like receptors and antimicrobial peptides during Eimeria praecox infection in chickens. Exp. Parasitol..

[71] Raqib R., Sarker P., Bergman P., Ara G., Lindh M., Sack D.A., Nasirul Islam K.M., Gudmundsson G.H., Andersson J., Agerberth B. (2006). Improved outcome in shigellosis associated with butyrate induction of an endogenous peptide antibiotic. Proc. Natl. Acad. Sci. USA.

[72] Liu P.T., Stenger S., Tang D.H., Modlin R.L. (2007). Cutting edge: Vitamin D-mediated human antimicrobial activity against Mycobacterium tuberculosis is dependent on the induction of cathelicidin. J. Immunol..

[73] Sarker P., Ahmed S., Tiash S., Rekha R.S., Stromberg R., Andersson J., Bergman P., Gudmundsson G.H., Agerberth B., Raqib R. (2011). Phenylbutyrate counteracts Shigella mediated downregulation of cathelicidin in rabbit lung and intestinal epithelia: A potential therapeutic strategy. PLoS One.

[74] Schauber J., Svanholm C., Termen S., Iffland K., Menzel T., Scheppach W., Melcher R., Agerberth B., Luhrs H., Gudmundsson G.H. (2003). Expression of the cathelicidin LL-37 is modulated by short chain fatty acids in colonocytes: Relevance of signalling pathways. Gut.

[75] Wang T.T., Nestel F.P., Bourdeau V., Nagai Y., Wang Q., Liao J., Tavera-Mendoza L., Lin R., Hanrahan J.W., Mader S. (2004). Cutting edge: 1,25-dihydroxyvitamin D3 is a direct inducer of antimicrobial peptide gene expression. J. Immunol..

[76] Gombart A.F., Borregaard N., Koeffler H.P. (2005). Human cathelicidin antimicrobial peptide (CAMP) gene is a direct target of the vitamin D receptor and is strongly up-regulated in myeloid cells by 1,25-dihydroxyvitamin D3. FASEB J..

[77] Schauber J., Dorschner R.A., Yamasaki K., Brouha B., Gallo R.L. (2006). Control of the innate epithelial antimicrobial response is cell-type specific and dependent on relevant microenvironmental stimuli. Immunology.

[78] Steinmann J., Halldorsson S., Agerberth B., Gudmundsson G.H. (2009). Phenylbutyrate induces antimicrobial peptide expression. Antimicrob. Agents Chemother..

[79] Schauber J., Iffland K., Frisch S., Kudlich T., Schmausser B., Eck M., Menzel T., Gostner A., Luhrs H., Scheppach W. (2004). Histone-deacetylase inhibitors induce the cathelicidin LL-37 in gastrointestinal cells. Mol. Immunol..

[80] Elloumi H.Z., Holland S.M. (2008). Complex regulation of human cathelicidin gene expression: Novel splice variants and 5'UTR negative regulatory element. Mol. Immunol..

[81] Schwab M., Reynders V., Loitsch S., Steinhilber D., Schroder O., Stein J. (2008). The dietary histone deacetylase inhibitor sulforaphane induces human beta-defensin-2 in intestinal epithelial cells. Immunology.

[82] Chakraborty K., Maity P.C., Sil A.K., Takeda Y., Das S. (2009). cAMP stringently regulates human cathelicidin antimicrobial peptide expression in the mucosal epithelial cells by activating cAMP-response element-binding protein, AP-1, and inducible cAMP early repressor. J. Biol. Chem..

[83] Cederlund A., Kai-Larsen Y., Printz G., Yoshio H., Alvelius G., Lagercrantz H., Stromberg R., Jornvall H., Gudmundsson G.H., Agerberth B. (2013). Lactose in human breast milk an inducer of innate immunity with implications for a role in intestinal homeostasis. PLoS One.

[84] Sunkara L.T., Achanta M., Schreiber N.B., Bommineni Y.R., Dai G., Jiang W., Lamont S., Lillehoj H.S., Beker A., Teeter R.G. (2011). Butyrate enhances disease resistance of chickens by inducing antimicrobial host defense peptide gene expression. PLoS One.

[85] Sunkara L.T., Jiang W., Zhang G. (2012). Modulation of antimicrobial host defense peptide gene expression by free fatty acids. PLoS One.

[86] Sunkara L.T., Zeng X., Curtis A.R., Zhang G. (2014). Cyclic AMP synergizes with butyrate in promoting beta-defensin 9 expression in chickens. Mol. Immunol..

[87] Bommineni Y.R., Dai H., Gong Y.X., Soulages J.L., Fernando S.C., Desilva U., Prakash O., Zhang G. (2007). Fowlicidin-3 is an alpha-helical cationic host defense peptide with potent antibacterial and lipopolysaccharide-neutralizing activities. FEBS J..

[88] Van Dijk A., Veldhuizen E.J., Kalkhove S.I., Tjeerdsma-van Bokhoven J.L., Romijn R.A., Haagsman H.P. (2007). The beta-defensin gallinacin-6 is expressed in the chicken digestive tract and has antimicrobial activity against food-borne pathogens. Antimicrob. Agents Chemother..

[89] Higgs R., Lynn D.J., Gaines S., McMahon J., Tierney J., James T., Lloyd A.T., Mulcahy G., O’Farrelly C. (2005). The synthetic form of a novel chicken beta-defensin identified in silico is predominantly active against intestinal pathogens. Immunogenetics.

[90] Xiao Y., Herrera A.I., Bommineni Y.R., Soulages J.L., Prakash O., Zhang G. (2009). The central kink region of fowlicidin-2, an alpha-helical host defense peptide, is critically involved in bacterial killing and endotoxin neutralization. J. Innate Immun..

[91] Derache C., Meudal H., Aucagne V., Mark K.J., Cadene M., Delmas A.F., Lalmanach A.C., Landon C. (2012). Initial insights into structure-activity relationships of avian defensins. J. Biol. Chem..

[92] McPhee J.B., Hancock R.E. (2005). Function and therapeutic potential of host defence peptides. J. Pept. Sci..

[93] Bowdish D.M., Davidson D.J., Hancock R.E. (2005). A re-evaluation of the role of host defence peptides in mammalian immunity. Curr. Protein Pept. Sci..

[94] Bowdish D.M., Hancock R.E. (2005). Anti-endotoxin properties of cationic host defence peptides and proteins. J. Endotoxin Res..

[95] Scott M.G., Dullaghan E., Mookherjee N., Glavas N., Waldbrook M., Thompson A., Wang A., Lee K., Doria S., Hamill P. (2007). An anti-infective peptide that selectively modulates the innate immune response. Nat. Biotechnol..

[96] Bommineni Y.R., Pham G.H., Sunkara L.T., Achanta M., Zhang G. (2014). Immune regulatory activities of fowlicidin-1, a cathelicidin host defense peptide. Mol. Immunol..

[97] Van Dijk A., Molhoek E.M., Veldhuizen E.J., Bokhoven J.L., Wagendorp E., Bikker F., Haagsman H.P. (2009). Identification of chicken cathelicidin-2 core elements involved in antibacterial and immunomodulatory activities. Mol. Immunol..

[98] Yu H.B., Kielczewska A., Rozek A., Takenaka S., Li Y., Thorson L., Hancock R.E., Guarna M.M., North J.R., Foster L.J. (2009). Sequestosome-1/p62 is the key intracellular target of innate defense regulator peptide. J. Biol. Chem..

[99] Mookherjee N., Lippert D.N., Hamill P., Falsafi R., Nijnik A., Kindrachuk J., Pistolic J., Gardy J., Miri P., Naseer M. (2009). Intracellular receptor for human host defense peptide LL-37 in monocytes. J. Immunol..

[100] Xiao Y., Dai H., Bommineni Y.R., Soulages J.L., Gong Y.X., Prakash O., Zhang G. (2006). Structure-activity relationships of fowlicidin-1, a cathelicidin antimicrobial peptide in chicken. FEBS J..

[101] Bhunia A., Mohanram H., Bhattacharjya S. (2009). Lipopolysaccharide bound structures of the active fragments of fowlicidin-1, a cathelicidin family of antimicrobial and antiendotoxic peptide from chicken, determined by transferred nuclear Overhauser effect spectroscopy. Biopolymers.

[102] Saravanan R., Bhattacharjya S. (2011). Oligomeric structure of a cathelicidin antimicrobial peptide in dodecylphosphocholine micelle determined by NMR spectroscopy. Biochim. Biophys. Acta.

[103] Landon C., Thouzeau C., Labbe H., Bulet P., Vovelle F. (2004). Solution structure of spheniscin, a beta-defensin from the penguin stomach. J. Biol. Chem..

[104] Tossi A., Sandri L., Giangaspero A. (2000). Amphipathic, alpha-helical antimicrobial peptides. Biopolymers.

[105] Dathe M., Wieprecht T. (1999). Structural features of helical antimicrobial peptides: Their potential to modulate activity on model membranes and biological cells. Biochim. Biophys. Acta.

[106] Nagaoka I., Kuwahara-Arai K., Tamura H., Hiramatsu K., Hirata M. (2005). Augmentation of the bactericidal activities of human cathelicidin CAP18/LL-37-derived antimicrobial peptides by amino acid substitutions. Inflamm. Res..

[107] Chen Y., Mant C.T., Farmer S.W., Hancock R.E., Vasil M.L., Hodges R.S. (2005). Rational design of alpha-helical antimicrobial peptides with enhanced activities and specificity/therapeutic index. J. Biol. Chem..

[108] Skerlavaj B., Gennaro R., Bagella L., Merluzzi L., Risso A., Zanetti M. (1996). Biological characterization of two novel cathelicidin-derived peptides and identification of structural requirements for their antimicrobial and cell lytic activities. J. Biol. Chem..

[109] Shin S.Y., Park E.J., Yang S.T., Jung H.J., Eom S.H., Song W.K., Kim Y., Hahm K.S., Kim J.I. (2001). Structure-activity analysis of SMAP-29, a sheep leukocytes-derived antimicrobial peptide. Biochem. Biophys. Res. Commun..

[110] Taylor K., Barran P.E., Dorin J.R. (2008). Structure-activity relationships in beta-defensin peptides. Biopolymers.

[111] Lehrer R.I., Lu W. (2012). alpha-Defensins in human innate immunity. Immunol. Rev..

[112] Wu Z., Hoover D.M., Yang D., Boulegue C., Santamaria F., Oppenheim J.J., Lubkowski J., Lu W. (2003). Engineering disulfide bridges to dissect antimicrobial and chemotactic activities of human beta-defensin 3. Proc. Natl. Acad. Sci. USA.

[113] Maemoto A., Qu X., Rosengren K.J., Tanabe H., Henschen-Edman A., Craik D.J., Ouellette A.J. (2004). Functional analysis of the alpha-defensin disulfide array in mouse cryptdin-4. J. Biol. Chem..

[114] Schroeder B.O., Wu Z., Nuding S., Groscurth S., Marcinowski M., Beisner J., Buchner J., Schaller M., Stange E.F., Wehkamp J. (2011). Reduction of disulphide bonds unmasks potent antimicrobial activity of human beta-defensin 1. Nature.

[115] Bommineni Y.R., Achanta M., Alexander J., Sunkara L.T., Ritchey J.W., Zhang G. (2010). A fowlicidin-1 analog protects mice from lethal infections induced by methicillin-resistant Staphylococcus aureus. Peptides.

[116] Molhoek E.M., van Dijk A., Veldhuizen E.J., Dijk-Knijnenburg H., Mars-Groenendijk R.H., Boele L.C., Kaman-van Zanten W.E., Haagsman H.P., Bikker F.J. (2010). Chicken cathelicidin-2-derived peptides with enhanced immunomodulatory and antibacterial activities against biological warfare agents. Int. J. Antimicrob. Agents.

[117] Molhoek E.M., van Dijk A., Veldhuizen E.J., Haagsman H.P., Bikker F.J. (2011). Improved proteolytic stability of chicken cathelicidin-2 derived peptides by d-amino acid substitutions and cyclization. Peptides.

[118] Zhou Y.S., Webb S., Lettice L., Tardif S., Kilanowski F., Tyrrell C., Macpherson H., Semple F., Tennant P., Baker T. (2013). Partial deletion of chromosome 8 beta-defensin cluster confers sperm dysfunction and infertility in male mice. PLoS Genet..

[119] Hancock R.E., Sahl H.G. (2006). Antimicrobial and host-defense peptides as new anti-infective therapeutic strategies. Nat. Biotechnol..

[120] Nijnik A., Madera L., Ma S., Waldbrook M., Elliott M.R., Easton D.M., Mayer M.L., Mullaly S.C., Kindrachuk J., Jenssen H. (2010). Synthetic cationic peptide IDR-1002 provides protection against bacterial infections through chemokine induction and enhanced leukocyte recruitment. J. Immunol..

[121] Klotman M.E., Chang T.L. (2006). Defensins in innate antiviral immunity. Nat. Rev. Immunol..

[122] Wilson S.S., Wiens M.E., Smith J.G. (2013). Antiviral mechanisms of human defensins. J. Mol. Biol..

[123] Nicholls E.F., Madera L., Hancock R.E. (2010). Immunomodulators as adjuvants for vaccines and antimicrobial therapy. Ann. N. Y. Acad. Sci..

[124] Yang J., Mao M., Zhang S., Li H., Jiang Z., Cao G., Cao D., Wang X., Zhang L. (2012). Innate defense regulator peptide synergizes with CpG ODN for enhanced innate intestinal immune responses in neonate piglets. Int. Immunopharmacol..

[125] Kovacs-Nolan J., Mapletoft J.W., Latimer L., Babiuk L.A., Hurk S. (2009). CpG oligonucleotide, host defense peptide and polyphosphazene act synergistically, inducing long-lasting, balanced immune responses in cattle. Vaccine.

[126] Kindrachuk J., Jenssen H., Elliott M., Townsend R., Nijnik A., Lee S.F., Gerdts V., Babiuk L.A., Halperin S.A., Hancock R.E. (2009). A novel vaccine adjuvant comprised of a synthetic innate defence regulator peptide and CpG oligonucleotide links innate and adaptive immunity. Vaccine.

[127] Zhang H.H., Yang X.M., Xie Q.M., Ma J.Y., Luo Y.N., Cao Y.C., Chen F., Bi Y.Z. (2010). The potent adjuvant effects of chicken beta-defensin-1 when genetically fused with infectious bursal disease virus VP2 gene. Vet. Immunol. Immunopathol..

[128] Soman S.S., Nair S., Issac A., Arathy D.S., Niyas K.P., Anoop M., Sreekumar E. (2009). Immunomodulation by duck defensin, Apl_AvBD2: *In vitro* dendritic cell immunoreceptor (DCIR) mRNA suppression, and B- and T-lymphocyte chemotaxis. Mol. Immunol..

[129] Li Y. (2011). Recombinant production of antimicrobial peptides in Escherichia coli: A review. Protein Expr Purif..

[130] Rotem S., Mor A. (2009). Antimicrobial peptide mimics for improved therapeutic properties. Biochim. Biophys. Acta.

[131] Campbell Y., Fantacone M.L., Gombart A.F. (2012). Regulation of antimicrobial peptide gene expression by nutrients and by-products of microbial metabolism. Eur. J. Nutr..

[132] Van der Does A.M., Bergman P., Agerberth B., Lindbom L. (2012). Induction of the human cathelicidin LL-37 as a novel treatment against bacterial infections. J. Leukoc. Biol..

